# Evaluation of ancestry from human skeletal remains: a concise review

**DOI:** 10.1080/20961790.2019.1697060

**Published:** 2019-12-23

**Authors:** Eugénia Cunha, Douglas H. Ubelaker

**Affiliations:** a National Institute of Legal Medicine and Forensic Sciences, Lisbon, Portugal;; b Laboratory of Forensic Anthropology, Centre for Functional Ecology, Department of Life Sciences, University of Coimbra, Coimbra, Portugal;; c Department of Anthropology, National Museum of Natural History, Smithsonian Institution, Washington, DC, USA

**Keywords:** Forensic sciences, forensic anthropology, ancestry estimation, skeletal remains

## Abstract

Ancestry assessment represents a major component of forensic anthropological analysis of recovered human remains. Interpretations of ancestry, together with other aspects of the biological profile, can help narrow the search of missing persons and contribute to eventual positive identification. Such information can prove useful to authorities involved in the identification and investigative process since many lists of missing persons have a reference to this parameter. Recent research has strengthened available methodologies involving metric, non-metric morphological as well as chemical and genetic approaches. This review addresses the new anthropological techniques that are now available, as well as the complex historical context related to ancestry evaluation.

## Introduction

Estimation of ancestry is important not only to assist identification directly, but also as a required precursor to estimating age, sex, stature and other attributes. Prior knowledge of ancestry for sex estimation, for example, can improve the accuracy of the estimation. Furthermore, missing lists do include a mention to ancestry. Since all identification is a comparative process, all four parameters, ancestry included, can lead to an exclusion.

Whenever ancestry is assessed, the methods actually being used both in research and casework seem to depend on the continent where they were developed. This paper approaches the methods that can be applied to assess the geographic origin, namely and mostly anthropological assessment (metric and non-metric methods) but also making some references to genetic and chemical methodologies, just to make clear that these later approaches are available and should be taken into account.

The published literature on ancestry issues in forensic anthropology is vast and complex but still leads to some confusion in the discipline regarding purpose and interpretation. For instances, there is still a general belief that only the skull works and that for the remaining skeleton there are only a few studies, which is not true. This concise review provides a needed overview of that literature to facilitate greater understanding of methods available and their application.

## History

Obviously, there has been a shift in thought about ancestry over time which has a reflection in the terminology which also suffered some changes. Those aspects, as well as classification systems involved in ancestry evaluation went through a “dark” period, which can be dated back to 18th century where typological and frequently racist attempts to categorize human variation occurred. The Swedish botanist Carolus Linnaeus (1707–1778) included humans in his broad binomial classification system of plants and animals [1]. Linnaeus gave humans the genus and species Homo sapiens. He also indicated that subdivisions based on geographical variation could be recognized. These subdivisions were classified as African (*H. afer*), American (*H. americanus*), Asian (*H. asiaticus*) and European (*H. europaeus*). Criteria for this typology were primarily based on impressions of behaviour and skin colour.

Subsequently, Johann Friedrich Blumenbach (1752–1840) extended the Linnaeus classification and added detail on features of head anatomy. Blumenbach added a new category for Malayan [2]. The classification systems of both Linnaeus and Blumenbach reflected attitudes of that time that such human variation was relatively fixed and static, as well as a religious perspective that a natural order of human variation existed and could be described.

Later attempts at classification varied extensively in regard to the breadth of group differentiation. Classifiers generally could themselves be classified as either lumpers or splitters, reflecting the number of groups identified [3]. In regard to American Indians, Morton and Hrdlička tended to agree with Linnaeus in a single group classification. In contrast, others (e.g. Retzius, Meigs, Virchow, Ten Kate, Dixon and Hooton) all recognized various subgroups within the general American Indian category [4].

With the development and acceptance of evolutionary theory among academics, scholars struggled to fit the earlier classification schemes into a more dynamic, modern perspective. Regional adaptation emerged as a guiding principle. Among the many examples of scholarship in this era, that of Carleton Coon stands out [5,6] in his various attempts to relate modern human diversity to the fossil record that existed at that time.

With augmented information on human variation, especially in regard to population genetics, the morphological boundaries of the old racial types became elusive. Data revealed a continuum of variation that was not clearly clustered into typological categories. In regards to the original criteria of Linnaeus, skin colour appeared to vary extensively in different regions of the world and the behaviour variables seem to reflect the attitudes and preconceptions of the classifiers rather than characteristics of populations. While the scientific basis of these original groupings gradually crumbled, the terminology persisted and became ingrained in public/folk classification.

The old racial concept of groups being static, pure and fixed gradually gave way to more dynamic, realistic views that recognized processes of gene flow and genetic variation within all groups and areas. Besides gene flow, dynamic adaptation (by natural selection), demography, namely population size, sexual selection and generic drift all have played a role in shaping the nowadays variation.

Quoting Roland B. Dixon, “a ‘race’ is not a permanent entity, something static, it is dynamic and is slowly developing and changing.” Additional definitions consist of “state of being one of a special people or ethnical stock” and reflecting use of the term in the general biological literature “a group or assemblage of organisms exhibiting general similarities but not sufficiently distinct from other forms to constitute a species” [7]. Although these definitions do not entirely reflect current views, they document the historical evolution of attitudes.

Although much of the racial terminology continued, embedded in public perceptions of variation, many anthropologists argued that the terms and underlying implied foundations had become toxic and subject to exploitation [8]. The influential T.D. Stewart argued that the need persisted to examine and document human variation but noted the evils of racism and that the word “race” had become problematic since it had different meanings to different people. Past ethnocentric classifications frequently reflected world-view and religious orientation and led to improper value judgements and racism. As noted by Boyd [9] race concepts, or even the lack thereof, varied extensively in different cultures.

With changing perspectives on the nature of population variation, terminology emerged as a major issue. Garn [10] used the terminology of geographic, local and micro races in an attempt to integrate then modern science into classification of human variation. Others refrained from using the term “race” in favour of ethnic group, breeding population and/or cline to describe human variation. Lasker [11] distinguished “biological race” from “social race” based on the extent to which differentiation depended upon biological attributes or groupings defined by ethnic or social factors.

The limited variation among humans at the genomic level has been discussed since Lewontin [12]. It is clear that the majority of the variation exists inside all human populations and only limited variation between populations [13]. In all, there is a limited amount of variation in humans that can be used to assist in generating an estimate of ancestry to assist with identification. But, above all, current efforts to discern ancestry from the skeleton aim to improve the likelihood of making a positive identification.

### Forensic terminology

Like biological anthropology in general, forensic anthropology has struggled with terminology related to the evaluation of ancestry. The goals of forensic anthropology include providing information (the biological profile) regarding an unidentified skeleton to assist authorities in attempts at identification. Since missing persons are frequently described using racial terminology, forensic anthropologists are guided to use that terminology as well. This effort is challenging in that the anthropologist needs to use terms that will be recognized and be useful in the search but also needs to avoid being labelled by colleagues as a 19th century taxonomist.

Two articles dealing with this dilemma in forensic anthropology have titles that succinctly summarize the issue. In 1992, Sauer [14] published an article with the provocative title “Forensic anthropology and the concept of race: if races don’t exist, why are forensic anthropologists so good at identifying them?” Kennedy followed in 1995 [15] with an article in *the Journal of Forensic Sciences* “But Professor, why teach race identification if races don’t exist?” Much of the substantive answer to these rhetorical questions was provided by Stewart back in 1979. In regard to the evaluation of race/ancestry in forensic anthropology, Stewart noted “from the stand point of forensic anthropology, it is necessary to categorize the skeletal remains of unknowns in terms that reflect racial reality as locally understood” [16]. This position largely reflects contemporary approaches to the topic. The goal is to avoid outdated typology but provide information and utilize language that will facilitate identification.

For all of these reasons, the use of the term “race” has diminished markedly in forensic anthropology publications, discussions, and forensic reports. Most forensic anthropologists (including the authors of this manuscript) prefer instead to discuss likely ancestry. Reports should focus on the likely ancestry of the examined individual or, alternatively, suggesting how this person likely would have characterized himself/herself or have been socially classified by the communities lived in. Such an approach provides the needed, useful information but avoids any suggestion of use of an outdated racial typology by the investigator. The biological information generated from anthropological analysis of a skeleton must be considered in judging how a person was regarded in terms of community definitions of race and ancestry. Nowadays, the recommended terms relating to the three main geographic groups are African, European and Asian. Specific cases may use targeted groups terminology as defined locally.

### Cultural/temporal approaches

It is useful to keep in mind that the goal of providing information on ancestry is to facilitate identification and allow searches of/for missing persons. As noted in the discussion above, even direct ancestry evaluation of human remains involves cultural/historical factors and local folk racial classifications. The categories themselves contain a social/historical component shaped by local culture and community standards of communication. The language employed in discussion and report writing should reflect local standards, i.e. guidelines, and be oriented to facilitate identification and not mislead [17,18].

Cultural data found on the skeleton also can provide direct evidence of ancestry. In most parts of the world cultural information can provide clues of the deep past and indicate that the recovered remains reflect archaeological contexts rather than modern forensic ones. Such information usually takes the form of associated artefacts that reveal culturally and temporally specific mechanisms of dating the remains.

Some cultural clues can be found directly on the skeleton itself. Although this is not a frequent finding in routine casework, it can be an added value when working in specific contexts such as, for instances, crimes against humanity in African countries. Classic examples include cultural modifications of the teeth and skulls that are known to reflect ancient specific practices. Dental modifications in antiquity are well-known in many parts of the world and reflect specific cultural practices. Incising and/or chipping of the teeth in particular patterns characterize various past American cultures, especially those concentrated in Mesoamerica [19]. These alterations include intentional filing of the teeth as well as drilling and the insertion of inlays [20,21]. Some African cultures filed their anterior teeth to form points, a custom that has also been reported historically from the Caribbean. Cybulski [22] describes alterations of the teeth produced by the wearing of decorative labrets among some Eskimo cultures. Eskimo dentitions also have revealed pressure chipping of the occlusal surfaces of teeth resulting from the use of teeth as tools to chew leather, crush bones and perform other stressful functions. The teeth of many past populations also present extreme patterns of occlusal wear that reflect ancient methods of food preparation. All of these conditions can suggest the individuals are ancient, not relating to modern periods of medico-legal interest.

Examples of intentional cranial modification (also called artificial deformation) can suggest antiquity as well. Such modifications have been recorded in many ancient cultures (Maya, Inca, among others). Many of these modifications are so culturally specific that both the time period and region/group can be identified with confidence.

Direct dating of recovered remains also allows ancient samples to be distinguished from modern ones. Such dating is closely linked to ancestry since detection of considerable antiquity likely suggests an ancestry different from the individuals represented in modern communities. Radiocarbon analysis represents the method of choice for such dating. Radiocarbon values with a percent modern value about 100 indicate the tissue sample formed after 1950 AD. Values below 100 indicate that radioactive decay is detected and the pre-1950 date can be estimated using the extent of that decay [23].

In some regions, cultural factors as described above can assist group identification. Such evaluation may prove forensically valuable in consideration of local population history. Similarly, information on medical procedures and related technology may facilitate population identification. This information is especially critical in the evaluation of unidentified migrants.

Finally, but not less important, the assessment of ancestry helps in using correct reference population for other methods that contribute to the biological profile: age, sex, and stature. This is the reason why some authors suggest this should be the first parameter to be assessed since the result will determine which methods should be used in the other three generic identification parameters.

## Anthropological assessment

In relation to ancestry in the anthropological assessment of the skeleton, there are two main approaches to be considered, non-metric and metric. The benefits and drawbacks of each of these approaches are worthwhile to mention: the less objective nature of the non-metric assessment is obvious. Non-metric analysis also requires more personal experience. For example, there is ambiguity in the evaluation of the nasal bridge, which can be classified as medium for one person and projected for another. Yet, it is also true that non-metric observations can capture much more information. We strongly recommend the use of both approaches, as follows.

No matter the type of approach, the accuracy of techniques is always dependent on validation studies. Until the method developed on the basis of a certain sample is applied in a different sample, the results cannot be validated. When choosing the methods one should always pay attention to double check whereas that method is recommended for the sample in question. Above all, it is important to ensure that there is an appropriate tool to distinguish ancestry that includes references from the population that the individual derives.

To infer about ancestry, the skull, in particular the mid-region of the face, is unanimously accepted as the most informative part of skeletal anatomy [24,25]. Therefore, we will mainly focus on cranial examination. All the procedures to allocate ancestry, no matter what statistical treatment is followed, focus on craniometrics or on non-metric traits based on the assumption that there is a significant cranial diversity [24]. However, it is obvious that there is a considerable overlap of the features, no matter their type. This is particularly true in the globalized world we are now living in due to the unprecedented admixture which can lead to very complex cases when attempting to distinguish ancestry from a skeleton, especially if we do not have a genomic assessment of predicted ancestry. Hence, the accuracy of ancestry estimation is hampered. It is important to bear in mind that cranial traits and measurements are always phenotypic features [26], partially determined by hereditability and influenced by the environment. Although there are polymorphisms that are quite distinctive of geographic regions, there isn’t a single trait that can be found only in a single population. The pattern of multiple traits offers a guide only to the most probable group of origin. Furthermore, some polymorphisms are highly useful as ancestry informative markers (mutations in LCT (lactase persistence)) [27,28].

### Non-metric approaches

**Figure 1.**
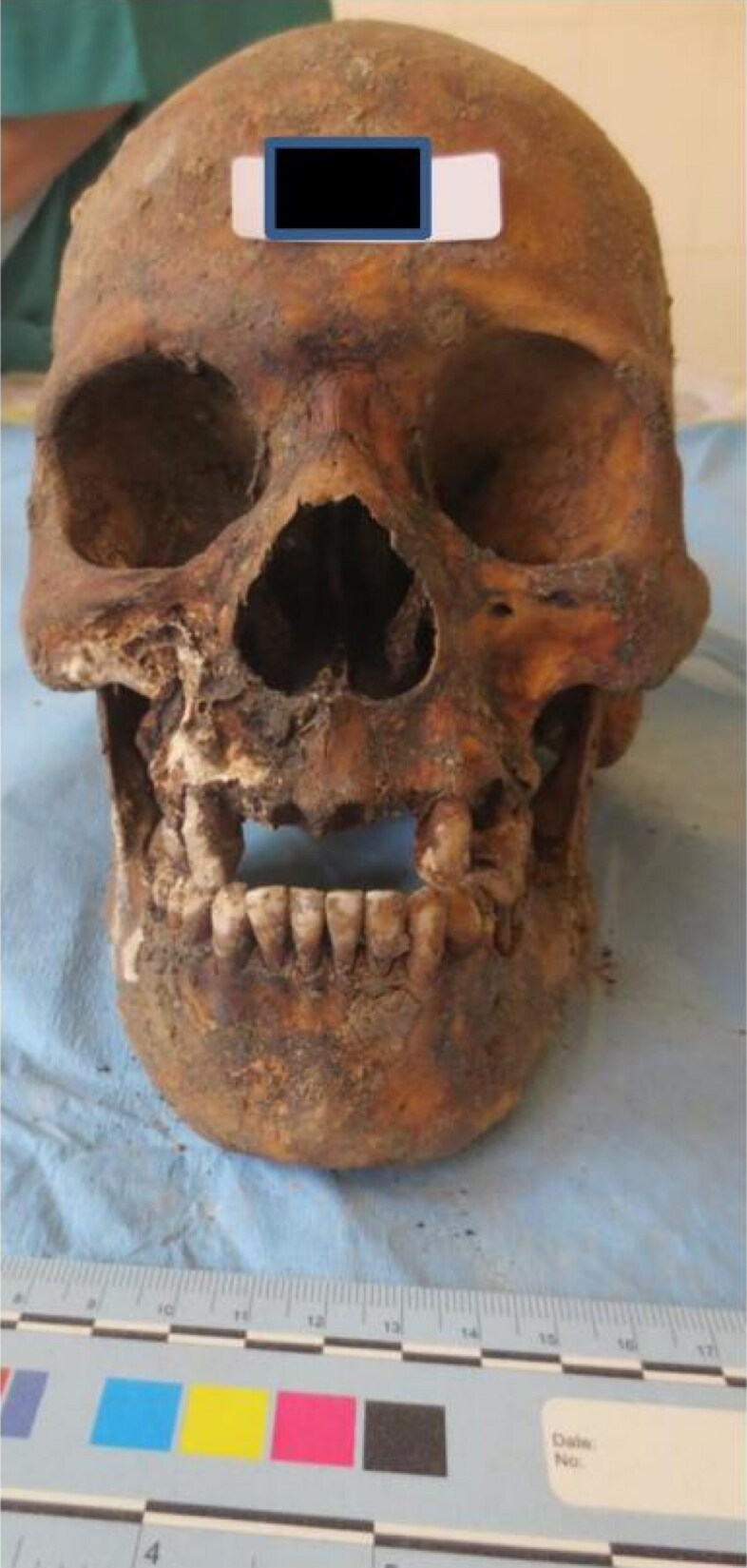
Example of a well preserved skull where ancestry was performed using both metric and non-metric approaches. The individual, with African ancestry, was positively identified. Note the large nasal aperture; the inexistence of anterior nasal spine, the inferior border of nasal aperture (incipient guttered); the large interorbital space; and the prognathism. All these features were paramount to the evaluation. Picture from the first author’s archive.

Within the non-metric approaches, two types of traits should be considered: morphoscopic ones, which evaluate the shape, and discrete traits, that are recorded as present or absent. The list of non-metric traits for the skull is particularly large; Hauser and De Stefano [29] describe more than 200. Among the morphoscopic features, suture shape, as well as palate shape are good examples; for the discrete traits, wormian bones and the metopic suture are among the most known ones. Hefner [30], in 2009, proposed a list to evaluate ancestry where the majority of the traits are located in the mid region of the face, in particular in the nasal area. Variation in the lower border of the nose, nasal aperture and the anterior nasal spine are among the key features to score (Figure 1).

Some years later, Hefner and Ousley [31] gave a step forward with the proposition of OSSA— Optimized Summed Scoring Attributes. Since no single trait or suite of traits accurately defines a population, there was a need to find a way to evaluate the threshold from which an individual could be considered as a member of a certain geographic group. OSSA quantifies the probability of a certain individual belonging to a given population [31]. The authors provided a score sheet where each trait is scored. The sum of all scores gives the ancestral group. Later, Navega and d’Oliveira Coelho [32] updated that score sheet by providing the posterior probability of a given individual belonging to a certain ancestral group.

The list of traits to be scored is now provided within some forensic anthropology books such as Işcan and Steyn [33] where very good illustrations of each trait, including its expression can be found. L’Abbé and collaborators [34] corroborate that it is possible to “arrive to a meaningful estimate of ancestry using non-metric traits” (pg. 212). A highlight should be given to the sutures shape that has been examined by some researchers [31] and that offers potential to differentiate between several groups.

The analysis of dental traits should always be considered as well in particular when the experts do not have a complete cranium or the morphological and/or metric analyses provide ambiguous results. Basically, non-metric dental approaches can be useful when only teeth are available for analysis and to support other methods. However, it should be bear in mind that dental studies are limited by teeth present and can have large error rates within their classification equations. Dental morphological characteristics have always been recognized as very informative in relation to ancestry and have a long history within dental anthropology. A recently edited volume on ancestry [35] includes two chapters on biological affinity estimation by dental traits. Two of the most known traits are Carabelli’s cusps and shoveling of incisors. Edgar [36] provides a good update on dental morphological estimation of ancestry. More recently, Scott and collaborators [37] using dental casts of the Arizona State University Dental Anthropology System (ASUDA) proposed *rASUDAS: a new Web-Based Application for Estimating Ancestry from Tooth Morphology* that uses crown and root morphology of the dentition. The reference sample is composed of 21 traits based on the ASUDA and represents approximately 30 000 individuals from seven geographic regions. The software is available at Osteomics’ platform [32].

To increase the power of non-metric traits to evaluate ancestry, the frequency of many more traits in additional populations around the world is needed. More information is required about the frequencies of well-known traits such as metopic suture, which is thought to be more common among Europeans [38], or the “Inca” bone, which is purportedly more frequent among South Americans [39]. A better knowledge on traits distribution should definitely be a priority in order to avoid problematic reliance on the experience of the observer.

### Metric approaches

The metric approach is more traditional and has more ancient roots. Craniometry has a long practice in both physical and forensic anthropology. It has the advantage of being more objective since each cranial measurement is well defined on the basis of likewise well-defined craniometrics points. Nevertheless, an index or simple ratio should not be used in forensic anthropology analysis for ancestry estimation [40] since more complex craniometrics methods offer greater accuracy.

What becomes critical is the way to combine the several cranial measurements and the selection of those that are more relevant. Linear discriminant analysis is one of the more used statistical approaches. This procedure was used in the North-American software FORDISC [41], which, on the basis of a maximum of 34 cranial and 39 postcranial measurements calculates discriminant function (DF). Essentially, an unknown individual will be compared with those represented in the database, that is, measurements of an unknown individual are compared with measurements of individuals with known ancestry in the database. This means that if the geographic region of the individual under analysis is not represented, the ancestral group cannot be found. Classification of an unknown individual is based on overall similarity. The accuracy is increased when sex estimation is performed by other means than the skull. The FORDISC database includes a forensic database as well as the famous Howells craniometrics series. Currently it is largely used in the US and because the database is composed largely of North American forensic cases, it works better there than in other geographic regions [25,42]. A good example of the specificity of FORDISC can be provided by the category of Hispanics, which is a socially constructed term where the word does not make reference to any biological feature but just to language and culture. A Portuguese individual can be easily classified as such which is inaccurate. Hence, when such terms are employed, it is important to recognize how they are defined locally.

Cranid is another software that enables the assessment of the skull's probable biological ancestry (in the broad geographical sense). On the basis of 29 measurements, the skulls are classified after comparison with 74 samples that include 3 163 skulls from around the world [43].

More recently, at the Laboratory of Forensic Anthropology in Portugal, Navega *et al.* [44] proposed a new forensic tool to evaluate ancestry on the basis of skeletal remains using a different statistical procedure. AncesTrees, which has now a database of nearly 3 000 individuals, can be considered a system to support the decision relying on a machine learning ensemble algorithm, random forest, to classify the human skull. In the ensemble learning paradigm, several models are generated and cojointly used to arrive at the final decision. The database used in AncesTrees is composed by 23 cranial measurements [44]. In the spreadsheet, the user enters the measurements taken from a skull and selects which ancestral groups should be included. The computer programme can be accessed freely at both http://lfa.uc.pt/ancestrees/ and at Osteomics website [32] (http://osteomics.com/AncesTrees). Both FORDISC and AncesTrees quantify the probability that a certain individual could belong to a given ancestral group, which is a major benefit in forensic sciences.

Lately, techniques of geometric morphometrics (GM) have allowed a good analysis of cranial shape through three-dimensional (3 D) coordinate data [45]. In all, GM is the statistical analysis of form based on Cartesian landmark coordinates. A software developed at North Carolina State University (NCSU) should be highlighted [45]. 3 D-iD is also a freely available software (www.3d-id.org/) which, besides ancestry, also allows sex assessment. To use 3D-iD, however, a digitizer is needed since most of the 3 D data are collected using digitizers that record the location of particular points in three dimensions. Worth to mention that this database is significantly increasing and that digital morphometrics is also being used to assess the shape of particular features, such as suture shape, as previously referred to, with ancestry proposals [46].

Noteworthy research is being conducted in South Africa where there are high levels of admixture. Nevertheless, despite that, linear discriminant analyses using craniometrics and, mostly, geometric morphometrics, have been able to identify group differences with high cross-validated accuracies (89%) [44].

Regarding postcranial methods, they are not only less investigated but also less used [47]. Although several postcranial bones are being searched, the femur is, by far, the most examined one. But new research is being published with promising results not only for the femur [47] but also for the tibia [48]. As with the skull, simple ratios like indexes should not be used to assess ancestry since they are influenced by physical stress and mobility.

### Other approaches

Ancestry analysis is one of the areas where the cooperation between forensic anthropologists and colleagues in genetics can be more fruitful [49]. When anthropological analysis raises a suspicion of a certain geographic area (Africa, Asia, or Europe), specific molecular markers, known as ancestry informative markers (AIMs) can have the answer. As forensic anthropologists, we only aim to provide some clues and call attention to the deep potential of genetics for ancestry estimation. For that purpose, a bone sample should be provided to the genetic lab, ideally a piece of the femur. Retrieving DNA from the skeleton has been incredibly improved during the last decades and nowadays it is possible to assess ancestry through targeted sequencing of very small quantities of DNA, including degrading samples.

Numerous molecular analyses using combinations of single nucleotide polymorphisms (SNPs), short tandem repeats (STRs), variable number of tandem repeats (VNTRs) or even certain insertions/deletions (INDELS) indicate strong molecular patterning in worldwide samples, allowing an accurate classification of groups, despite large amounts of within region variation [50]. For that purpose, population genetics studies are essential, since we cannot find the origin of one person in a database if the respective geographic region is not represented. As stated by Callaway [51], “You can’t tell someone they can trace ancestry to a certain region if that region has never been studied.” Furthermore, SNIPs have been allowing a differentiation, without error among African, Asian and European groups [52] proving that a good panel of ancestry informative SNPs can provide very good estimates. Also noteworthy that DNA analysis of AIMs and physical trait markers from biological stains can also help provide investigative leads in cases without suspects. Some specific markers of the populations are searched (SNP) that can suggest physical traits. Yet, theoretically, STRs have more power to identify [53,54]. The interface of the performances of genetics and craniometrics is a good example of the interdisciplinary nature of this parameter [55].

Furthermore, efforts like the HGDP, HapMap, and 1 000 Genomes Projects (1KGP) have elucidated the (limited) variation that exists within and between human populations. Studying these differences have also helped reveal the adaptiveness of some mutations to specific environments that may also be revealed in phenotypic variation (pigmentation, nose shape…). These databases are useful as references when assessing ancestry composition from the genome [56–58].

In practical casework, it is nowadays routine to send a human bone or teeth to genetic analyses and to cross and discuss the results in conjunction with the genetician. This procedure is thus recommended.

Spatially distributed isotope datasets can also be used to address the question of biological affinity [59]. Stable isotope analysis is an effective geolocation tool since isotopes provide a record of movement and eating habits of an individual throughout life. Saskia Ammer’s project [60] on that provides a good example of a societal benefit derived from ancestry estimation. Isotope analysis can be especially useful with identification of likely migrants.

Secondary ancestry identifiers also can be used not only to create a suspicion of ancestry but also to corroborate a hypothesis. Above all, the assessment of the geographic origin is a holistic approach in which perspective from different disciplines and datasets are important. For example, clothes, labels of clothes and personal belongings can provide some guidance. Within these secondary ancestry indicators, epidemiological data on some bone diseases may provide some clues since some pathological conditions are more frequent in some regions of the world.

## Reporting ancestry

As the report is an important component in a forensic case, the way ancestry is reported is likewise paramount. It is important to clearly state how ancestry was evaluated, namely, which bones/anatomical areas were used. The methods applied should also be clearly indicated. Equally important when reporting ancestry is to include the accuracy of the method.

Anthropologists should not “overstep” with their ancestral classification of the remains (e.g. classifying a skeleton as being of European ancestry when only postcranial remains are available for analysis), and should use “probable” or caveat the ancestral classification when appropriate. Furthermore, there are always a considerable number of cases in which the final result is indeterminate. In the use of AncesTrees, FORDISC, Ossa, genomic assessment and 3D-iD, it is possible to quantify the statistical probability of belonging to a certain population group. Otherwise, terms like possible, probable, compatible and consistent, or indeterminate are usually employed [61].

Since databases used by law enforcement organizations and missing persons lists make reference to ancestry, this parameter may allow an exclusion. If the recovered skeleton suggests an African origin and all the individuals in a given data basis are European, an exclusion can be hypothesized, that is, we can state that most probably there is no match with any of the missing list individuals. However, it should be recognized that many populations are poorly represented in the published literature regarding skeletal morphology. Hence, caution is called for. More comprehensive databases are needed for missing persons to strengthen identification efforts.

## Final considerations

Among the major four parameters of the biological profile, ancestry is the least applied since there are still some practitioners in some countries who don’t do it. Indeed, there are still many forensic anthropologists who simply do not evaluate ancestry despite the missing persons lists always make reference to the geographic origin of the disappeared. Unquestionably, this parameter remains controversial [62]. We argue that currently, updated and critical literature can be consulted on the subject [63], and objective guidelines and softwares are now available, some of which are reviewed in this article. However, with respect to the use of software and mathematical formulae in general, the expert should always verify whether the appropriate geographic region is represented in the databases. The quantification of the results and establishment of statistical probability are of utmost importance for forensic anthropology. Moreover, the validation of the methods worldwide is also critical. The existence of identified collections that include the knowledge of the geographic origin, as it is the case of the Brazilian collections [64], should be highlighted since they offer opportunities for improvement in the accuracy of ancestry assessment.

The literature clearly shows that the majority of forensic anthropology ancestry research has focused extensively on the skull but bones such as the femur and the tibia also provide useful information. Above all, a holistic approach, including anthropology, genetics, genomic, chemistry, and other disciplines, offers opportunities not only for exclusion but also to generate useful information to assess ancestry from recovered human remains.
